# Impact of Adenosine Analogue, Adenosine-5′-N-Ethyluronamide (NECA), on Insulin Signaling in Skeletal Muscle Cells

**DOI:** 10.1155/2021/9979768

**Published:** 2021-06-26

**Authors:** Mansour Haddad

**Affiliations:** Faculty of Pharmacy, Philadelphia University, Amman, Jordan

## Abstract

**Materials and Methods:**

Rat L6 skeletal muscle cells were cultured in 25 cm^2^ flasks. These differentiated cells were treated, and then, quantitative reverse transcription-polymerase chain reaction (qRT-PCR) (probe-based) was used to measure the relative mRNA expression level for metabolic, inflammatory, and nuclear receptor genes including peroxisome proliferator-activated receptor gamma (PGC-1*α*), carnitine palmitoyl transferase 1 beta (CPT1B), long-chain acyl-CoA de hydrogenase (LCAD), acetyl-CoA carboxylase beta (ACC*β*), pyruvate dehydrogenase kinase 4 (PDK4), hexokinase II (HKII), phosphofructokinase (PFK), interleukin-6 (IL-6), and nuclear receptor subfamily 4, group A (NR4A) at different treatment conditions.

**Results:**

Adenosine-5′-N-ethyluronamide (NECA), a stable adenosine analogue, significantly stimulate inflammatory mediator (IL-6) (*p* < 0.001) and nuclear receptors (NR4A) (*p* < 0.05) and significantly modulate metabolic (PFK, LCAD, PGC-1*α*, and CPT1B) gene expressions in skeletal muscle cells (*p* < 0.05, *p* < 0.05, *p* < 0.001, and *p* < 0.01, respectively). This present study shows that there is a noteworthy crosstalk between NECA and insulin at various metabolic levels including glycolysis (HKII), fatty acid oxidation (ACC*β*), and insulin sensitivity (PDK4).

**Conclusions:**

A novel crosstalk between adenosine analogue and insulin has been demonstrated for the first time; evidence has been gathered in vitro for the effects of NECA and insulin treatment on intracellular signaling pathways, in particular glycolysis and insulin sensitivity in skeletal muscle cells.

## 1. Introduction

Numerous experimental data indicate that adenosine can affect insulin-mediated metabolic processes in skeletal muscle cells [1–3]. It has been reported that the addition of adenosine receptor agonists such as GR79236 or antagonists such as 8-phenyltheophylline to the incubation medium can induce remarkable changes in the insulin sensitivity of isolated rat soleus muscle [1, 2, 4–7]. While a potential functional role for the adenosine modulation of metabolism in various insulin-sensitive tissues has been demonstrated, there appears to be uncertainty over whether adenosine activation potentiates or inhibits metabolism in the skeletal muscle tissue [1, 2, 4–9]. Furthermore, information related to the adenosine modulation of insulin regulation of glucose uptake and metabolism in humans, particularly at the level of skeletal muscle, is incomplete. Several studies have also shown that adenosine modulates glucose utilization pathways in the skeletal muscle [1, 2, 4–7, 10–15]. However, these findings are considered to be controversial in increasing or decreasing glucose utilization/metabolism.

Although adenosine has clearly been shown to potentiate insulin's stimulatory actions on glucose uptake in adipose tissue and the heart [16–21], adenosine modulation of carbohydrate metabolism/glucose uptake in skeletal muscle is still very controversial in increasing or decreasing glucose uptake, and to date, conflicting results have been published [1, 22]. Similarly, reports on adenosine role in diseases associated with insulin resistance, such as diabetes mellitus and obesity, are equally conflicting regarding the effect of adenosine on regulating insulin resistance/sensitivity [8, 23–25]. Adenosine is assumed to play a role in regulating glucose transport, as well as other metabolic and inflammatory processes, in various insulin-sensitive tissues, such as adipose tissue and skeletal muscle. Within this pattern, the potential impact of adenosine analogues on insulin sensitivity in the gene expression regulation of metabolic and inflammatory processes deserves further investigation.

Insulin may have an impact on the skeletal muscle tissue in the body. Its effect on tissue might be through alteration in the inflammation and metabolism level [26]. Insulin signaling regulates numerous pathways in the skeletal muscle that contributes to glucose metabolism [27]. Indeed, insulin resistance is correlated with various inflammatory responses which play a crucial role in the decline of insulin sensitivity [26]. Significant progress in understanding the development of insulin resistance has been previously investigated; however, the precise molecular mechanisms responsible for insulin resistance, particularly in the skeletal muscle, still remain incompletely understood.

It is unclear whether or not changes in the concentration of adenosine in skeletal muscle are responsible for the modulatory action of adenosine on the sensitivity of different metabolic/inflammatory key genes to insulin. Therefore, it is important to investigate the effects of adenosine analogue (NECA) on the sensitivity of key metabolic/inflammatory genes (IL-6, PDK4, HKII, PFK, LCAD, PGC-1*α*, CPT1B, ACC*β*, and NR4A) to insulin in skeletal muscle cells.

## 2. Materials and Methods

### 2.1. Materials

Fetal bovine serum (FBS) was obtained from Capricorn Scientific, USA. Horse serum and insulin were purchased from Sigma Chemical Company, Germany. TRIzol reagent and charcoal stripped fetal bovine serum were obtained from Applied Biosystems, USA; and dimethyl sulphoxide (DMSO) reagent was obtained from Santa Cruz, USA. Ham-F 10 was supplied by PAA Company, USA. Dulbecco's Modified Eagle Medium (DMEM) was purchased from Caisson, USA. Adenosine-5′-N-ethyluronamide (NECA) was purchased from Tocris Bioscience, UK. RNeasy Mini Total RNA Purification kits and RNase-Free DNase set were obtained from Qiagen, Germany. Maxima probe quantitative polymerase chain reaction (qPCR) Master Mix (2X) and Thermo Scientific RevertAid First Strand complementary DNA (cDNA) Synthesis Kits were brought from Thermo Scientific Company, USA.

### 2.2. Cell Culture

Rat L6 myoblast cell line (originally obtained from the American Type Culture Collection (USA)) was incubated at 37°C and 5% CO_2_ in Dulbecco's Modified Eagle Medium- (DMEM, Sigma, UK) high glucose (4500 mg/L), 10% (*v*/*v*) heat-inactivated FBS, 1% L-glutamate, and 1% penicillin/streptomycin. The culture was performed to instruction from the American Type Culture Collection (USA).

**Figure 1:**
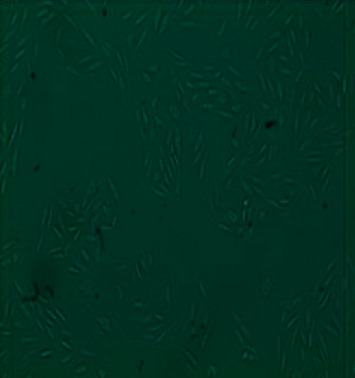
Representative myoblasts derived from passage number 7; myoblasts taken after 1 day seeding into 25 cm^2^ (10x).

**Figure 2:**
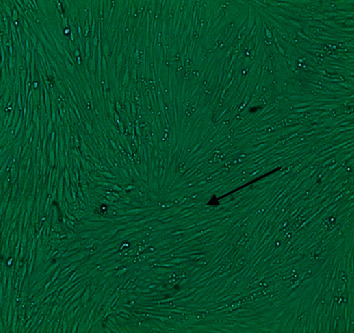
Representative myotubes derived from passage number 7; myotubes taken (Ham-F10, 1% P/S) after 7 days of starvation (10x).

Rat L6 myoblast cell lines were grown to confluence in 25 cm^2^ flasks. The cells were grown for around 14 days, checking for differentiation (to allow myotube formation) and changing the medium 2–3 times per week (every other day), according to the protocol mentioned in [28] (Figure 1). Once 70-90% confluent myotubes (approximately 2 weeks in culture) occurred, cells were serum-starved (incubated in Ham-F 10 medium alone) for 7 days. Then, cells (Figure 2) were treated for 1 h with vehicle (0.1% DMSO) and for 10 min with insulin 100 nM (cells were pretreated with NECA for 1 h prior to the addition of insulin) [15, 28]. After that, cells were washed with ice-cold PBS, then lysed with TRIzol (Invitrogen product name) (2 mL per flask).

### 2.3. Total RNA Extraction and Reverse Transcription

Rat L6 skeletal muscle cells were collected in 2 mL of ice-cold TRIzol (Applied Biosystems, USA), and total RNA was extracted from those cells according to the manufacturer's instructions of TRIzol reagent. Total RNA clean-up and on-column DNAse digestion were performed using RNeasy purification columns. RNA concentration and purity were quantified using a spectrophotometer (JENWAY Genova Nano). Reverse transcription was carried out from 500 ng total RNA using RevertAid First Strand cDNA Synthesis.

### 2.4. Taqman Quantitative Real-Time PCR

A relative standard curve method based on Taqman quantitative real-time PCR (qRT-PCR) was used to quantify gene expression. Taqman primers and probes (which were obtained from by Integrated DNA Technologies, Inc., USA.) were designed using Primer Express software (Applied Biosystems, USA) (Table 1). Assays were performed in triplicate according to the protocol mentioned in [28]. The threshold values for each triplicate were averaged, and the quantification of expression of each gene relative to reference gene determined using the standard curve method.

### 2.5. Statistical Analysis

Statistical differences among treatments were assessed using one-way ANOVA with the Tukey test. Statistical significance was accepted at a 5% level. Analysis was performed using GraphPad Prism, version 5.03 (GraphPad Software Inc.). Results are presented as the means ± SEM.

## 3. Results

### 3.1. NECA Stimulated IL-6, NR4A, PGC-1*α*, PFK, and LCAD mRNA Gene Expression in Rat Skeletal Muscle Cells

**Figure 3:**
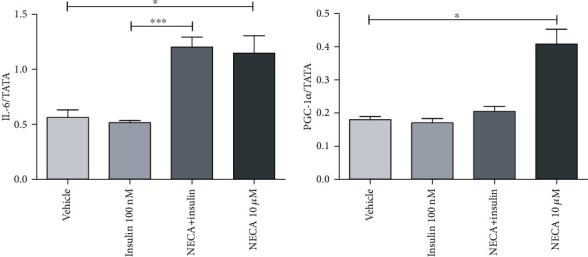
Effects of adenosine-5′-N-ethyluronamide (NECA), insulin on IL-6 and PGC-1*α* messenger RNA (mRNA) gene expression in rat L6 skeletal muscle myotubes using charcoal serum (70-90% confluent) starved for 7 days and then stimulated for 1 hour with NECA and then for 10 minutes with insulin (100 nM). IL-6 mRNA levels were measured relative to TATA-BOX using qRT-PCR; stimulation was performed with vehicle (0.1% dimethyl sulphoxide), NECA (10 *μ*M), and insulin (100 nM); data are represented as the means ± SEM of at least three independent experimental groups. ∗ denotes *p* < 0.05 and ∗∗∗ denotes *p* < 0.001. Data are analysed using a one-way ANOVA test followed by a Tukey test.

**Figure 4:**
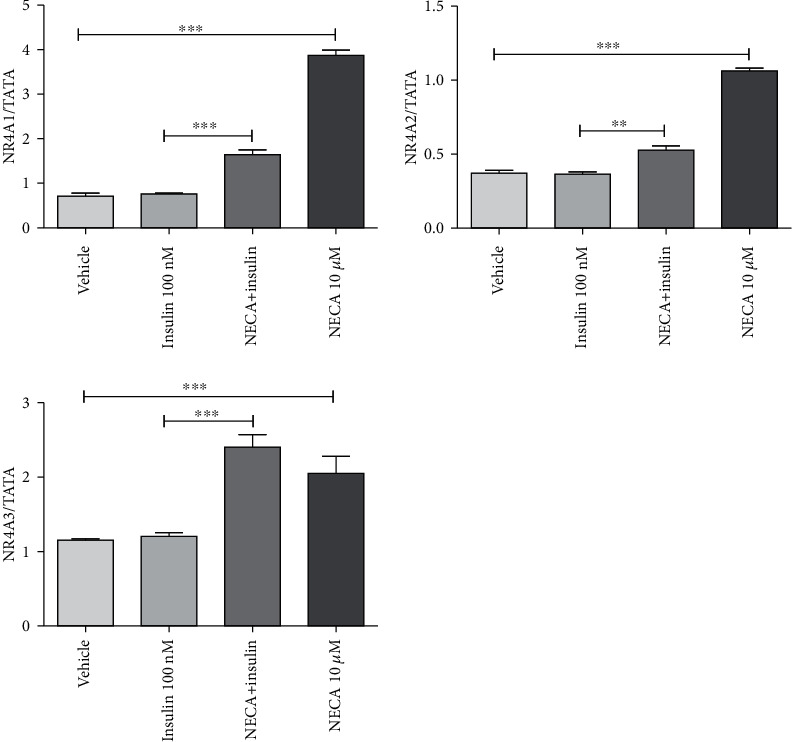
Effects of adenosine-5′-N-ethyluronamide (NECA), insulin on NR4A1, NR4A2, and NR4A3 messenger RNA (mRNA) gene expression in rat L6 skeletal muscle myotubes using charcoal serum (70-90% confluent) starved for 7 days and then stimulated for 1 hour with NECA and then for 10 minutes with insulin (100 nM). IL-6 mRNA levels were measured relative to TATA-BOX using qRT-PCR; stimulation was performed with vehicle (0.1% dimethyl sulphoxide), NECA (10 *μ*M), and insulin (100 nM); data are represented as the means ± SEM of at least three independent experimental groups. ∗∗ denotes *p* < 0.01 and ∗∗∗ denotes *p* < 0.001. Data are analysed using a one-way ANOVA test followed by a Tukey test.

**Figure 5:**
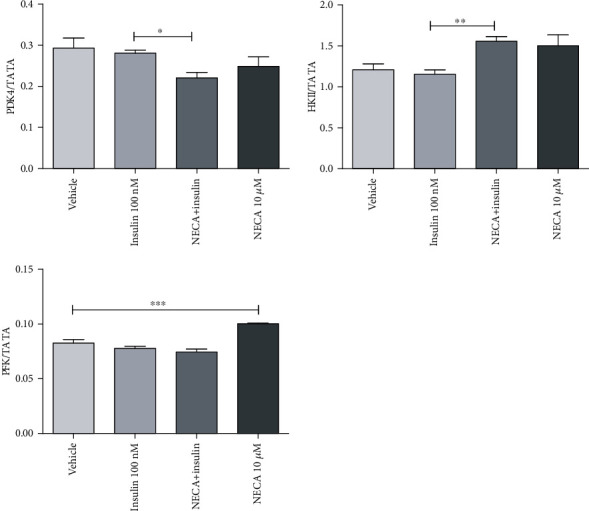
Effects of adenosine-5′-N-ethyluronamide (NECA), insulin on PDK4, HKII, and PFK messenger RNA (mRNA) gene expression in rat L6 skeletal muscle myotubes using charcoal serum (70-90% confluent) starved for 7 days and then stimulated for 1 hour with NECA and then for 10 minutes with insulin (100 nM). IL-6 mRNA levels were measured relative to TATA-BOX using qRT-PCR; stimulation was performed with vehicle (0.1% dimethyl sulphoxide), NECA (10 *μ*M), and insulin (100 nM); data are represented as the means ± SEM of at least three independent experimental groups. ∗ denotes *p* < 0.05, ∗∗ denotes *p* < 0.01, and ∗∗∗ denotes *p* < 0.001. Data are analysed using a one-way ANOVA test followed by a Tukey test.

**Figure 6:**
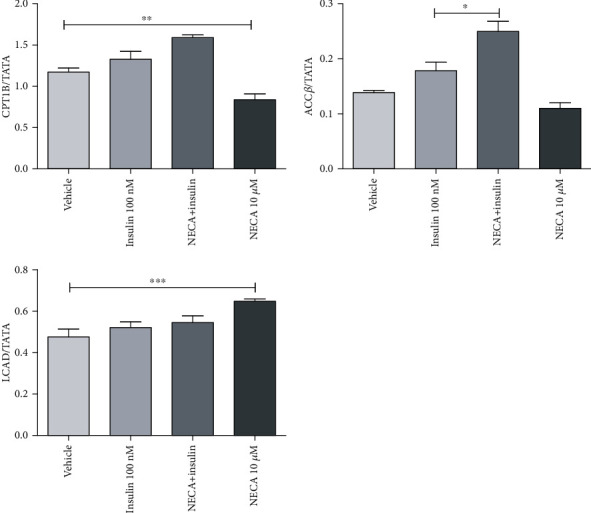
Effects of adenosine-5′-N-ethyluronamide (NECA), insulin on CPT1B, ACC*β*, and LCAD messenger RNA (mRNA) gene expression in rat L6 skeletal muscle myotubes using charcoal serum (70-90% confluent) starved for 7 days and then stimulated for 1 hour with NECA and then for 10 minutes with insulin (100 nM). IL-6 mRNA levels were measured relative to TATA-BOX using qRT-PCR; stimulation was performed with vehicle (0.1% dimethyl sulphoxide), NECA (10 *μ*M), and insulin (100 nM); data are represented as the means ± SEM of at least three independent experimental groups. ∗ denotes *p* < 0.05, ∗∗ denotes *p* < 0.01, and ∗∗∗ denotes *p* < 0.001. Data are analysed using a one-way ANOVA test followed by a Tukey test.

Incubation of one-week starved L6 skeletal muscle cells (Figure 2) with 10 *μ*M NECA upregulated IL-6, NR4A1, NR4A2, NR4A3, PGC-1*α*, PFK, and LCAD. The mRNA gene expression of IL-6 showed a significant increase (*p* < 0.001) of almost 2.3 times the normal expression (Figure 3). Other genes such as NR4A1, NR4A2, and NR4A3 indicated significant (*p* < 0.05) increases of almost 5.9, 2.9, and 2.5 times, respectively, in the gene expression (Figure 4). The expression of PGC-1*α* was also increased 2.5 times the normal expression (*p* < 0.05) (Figure 3). Both PFK and LCAD depicted 1.3 times higher expression. The increases were quite significant with the *p* values being (*p* < 0.05) for PFK (Figure 5) and (*p* < 0.001) for LCAD (Figure 6).

### 3.2. NECA Inhibited CPT1B mRNA Gene Expression in Skeletal Muscle Cells

Incubation of one-week starved L6 skeletal muscle cells with 10 *μ*M NECA inhibited the expression of CPT1B (0.76-fold change compared to vehicle) significantly (*p* < 0.05) (Figure 6).

### 3.3. NECA Did Not Alter PDK4, HKII, and ACC*β* mRNA Gene Expression in Skeletal Muscle Cells

Genes such as PDK4, HKII, and ACC*β* did not show any alterations in their expression patterns (Figures 5 and 6). The mRNA gene expressions of these genes were unaffected even after incubation of one-week starved L6 skeletal muscle cells with NECA (10 *μ*M) for 1 h.

### 3.4. Insulin Did Not Modulate the Inflammatory, Nuclear Receptor, and Metabolic Gene Expression

The mRNA gene expression of IL-6, NR4A, PGC-1*α*, CPT1B, LCAD, ACC*β*, PDK4, and PFK was unaffected after incubation of one-week starved L6 skeletal muscle cells with insulin (100 nM), resulting in an insulin resistance in this model.

### 3.5. Adenosine Analogue, NECA, Modulated the Action of Insulin in Skeletal Muscle Cells

Incubation of one-week starved L6 skeletal muscle cells with 100 nM of insulin for 10 min (pretreated by NECA (10 *μ*M) for one hour) increased significantly (*p* < 0.05) mRNA gene expression of IL-6 (around 2.4-fold change compared to insulin), similar to the effect of NECA. In the myotubes, NECA- (10 *μ*M) evoked elevation in IL-6 mRNA expression was unaltered in the presence of insulin (100 nM) for 10 min (insulin was added after NECA was treated), indicating that there is no crosstalk between adenosine analogue and insulin on this inflammatory mediator (IL-6) (Figure 3).

Insulin alone or NECA alone had no effect on PDK4. In response to sequential NECA, insulin treatment (cells were pretreated with NECA for 1 h prior to the addition of insulin), PDK4 decreased significantly (*p* < 0.001) (10 min insulin), 0.8-fold compared to insulin, suggesting NECA and insulin interact to decrease cellular PDK4 expression, resulting in potential improved glucose utilization and insulin sensitivity (Figure 5). NECA and insulin together showed 1.3-fold significant increase (*p* < 0.01) in the expression of HKII compared to insulin, suggesting an improvement in the glycolysis process (Figure 5).

Insulin alone had no effect on PFK and LCAD mRNA expression in the skeletal muscle. NECA induced an around 1.3-fold significant increase (*p* < 0.05) in PFK and LCAD compared to vehicle. NECA and insulin together did not induce any significant alteration in the expression level of PFK and LCAD (Figures 5 and 6, respectively).

In response to NECA alone (but not insulin alone), PGC-1*α* was increased significantly (*p* < 0.01) after 1 h treatment (2.5 fold change compared to vehicle). However, in response to sequential NECA, insulin treatment, NECA, and insulin together could not bring about any significant change in the expression of PGC-1*α* (Figure 3).

Insulin alone had no effect on CPT1B and ACC*β*. In response to NECA alone, expression of CPT1B decreased significantly (*p* < 0.01) after 1 h treatment (0.76-fold change compared to vehicle), while in response to sequential NECA, insulin treatment, NECA, and insulin together, there was no alteration in gene expression. However, in response to NECA alone, there was no change in expression of ACC*β*, while in response to sequential NECA, insulin treatment, NECA and insulin together did show around 1.4-fold significant increase (*p* < 0.01) in ACC*β* levels, indicating the importance of this crosstalk regarding lipid metabolism (Figure 6).

Insulin alone had no effect on NR4A1, NR4A2, and NR4A3 gene expression. There is a difference in the extent of expression for NR4A between two conditions (NECA compared to NECA and insulin together), in particular for NR4A1. The fold change in response to NECA was around 5.9, 2.9, and 2.5 compared to vehicle for NR4A1, NR4A2, and NR4A3, respectively, while the fold change in response to NECA and insulin was 1.73, 1.4, and 2.0 compared to insulin for NR4A1, NR4A2, and NR4A3, respectively, indicating the particular potential importance of this crosstalk regarding NR4A1 (Figure 4).

## 4. Discussion

The major novel findings in the present study are as follows: (i) stable adenosine analogue, NECA, increases gene expressions involved in inflammation (*IL-6*), nuclear receptors (*NR4A*; particularly *NR4A1*), glycolysis (*PFK*), and energy metabolism (*PGC-1α*), while also modulating gene expression involved in fatty acid transport and oxidation (*LCAD* and *CPT1B*); (ii) NECA and insulin together decrease gene expression involved in insulin sensitivity (*PDK4*) and increase gene expression implicated in glycolysis (*HKII*) and fatty acid oxidation (*ACCβ*); and (iii) insulin alone had no effect on the gene expression in one week starved cells.

These data suggest that the presence of adenosine in skeletal muscle is required for insulin to modulate metabolic effects and that these effects of adenosine may occur via a receptor-mediated or an uptake-mediated event. Essentially, these data demonstrate that *in vitro*, adenosine-mediated activity in skeletal muscle is required for insulin to modulate functionally metabolic molecular signaling. It is therefore possible that the endogenous level of adenosine, potentially acting at its receptors, is required for insulin action in skeletal muscle.

This present study has demonstrated that one-week starved (media without FBS) skeletal muscle cells *in vitro* exhibited a marked resistance to the effect of insulin on metabolic and inflammatory genes: this further confirms the findings reported by many researchers who found that starvation induced insulin resistance [29, 30]. Insulin resistance (reduced insulin sensitivity) is associated with many conditions including a high-fat diet, lipid infusion, type 2 diabetes, obesity, and starvation [31]. The development of insulin resistance following starvation is a normal physiological response in which starvation induces a real change in the ability of the peripheral tissues to utilize glucose [32]. Therefore, starvation may well be considered to be one of the earliest forms of insulin resistance in man. Various studies have produced controversial evidences about the mechanism of starvation-induced insulin resistance [30, 33]. Of note, it is proposed that the development of insulin resistance during starvation may be linked to the potential alteration of metabolic and inflammatory functional genes. The present study agrees with the majority of the literature in that insulin did not alter the expression of metabolic, nuclear, and inflammatory genes investigated in this study, thereby indicating that insulin resistance was induced during the one-week starvation period. Insulin resistance is a common finding associated with conditions such as obesity and diabetes. It is considered to arise either from insulin receptor abnormalities or deficiencies, from poorly defined “postreceptor defects,” or from a combination of problems [34]. The findings presented in this study suggest a further potential mechanism by which insulin sensitivity, at least in the skeletal muscle, could be altered, i.e., via abnormalities in adenosine concentration.

A novel relationship between NECA and IL-6 has been introduced in the present study; IL-6 upregulation was induced by NECA in rat skeletal muscle cells. However, no crosstalk between NECA and insulin was observed in terms of inflammation, IL-6. This novel finding suggests a signal transduction gene expression mechanism whereby IL-6 is even regulated by NECA in skeletal muscle cells. Therefore, adenosine analogue may act as a proinflammatory mediator in this tissue. In the present study, the effects of the adenosine receptor agonist, NECA, on the skeletal muscle derived from rats in which insulin sensitivity (downregulation of *PDK4*) are increased supporting the view that an increase in the concentration of adenosine improves the sensitivity of glycolysis to insulin (upregulation in *HKII*). Thus, insulin sensitivity is potentially improved by the adenosine analogue, NECA, in this study. The findings presented here clearly support a potential novel function for adenosine in skeletal muscle cell modification of insulin sensitivity.

Hue et al. [35] clearly clarified the glucose and fatty acid cycle in which a decrease in glucose uptake and utilization in rat heart and diaphragm *in vitro* is a result of an increase in lipid availability in those tissues. As the skeletal muscle contains endogenous triglyceride and a reciprocal cycle is available between glucose and fatty acid in skeletal muscle tissue according to Hue et al. [35], a decrease in lipolysis (a decrease in the rate of fatty acid oxidation) by NECA may result in a reciprocal increase in the rate of glycolysis and glucose oxidation. In the present study, short-term treatment of one-week starved skeletal muscle cells with NECA has been shown to decrease the expression of genes involved in fatty acid transport CPT1B, indicating that potentially decreased fatty acid influx into muscle during exposure of the cells to high concentrations of NECA is accompanied by an adaptive decrease in gene expression. Moreover, short-term treatment of both NECA and insulin of the same starved muscle cells has been shown to increase the expression of ACC*β*, which might allow the cell to switch faster from fatty acid to glucose oxidation (through increase in levels of malonyl CoA and consequently inhibition of CPT1B) without compromising the overall ability of the cell to oxidize fatty acids.

It is noteworthy that mRNA expression levels may reflect signaling protein transcription and turnover and may represent activation or even more global insulin sensitivity in skeletal muscle. However, further research should be performed later to assess protein expression and activation (phosphorylation), and a further work including some functional measurements as glucose uptake should be performed to draw more supportive conclusions on insulin signaling. Moreover, a further work should also be performed to assess more crucial genes linked with insulin signaling in skeletal muscle including (IR, IRS, AKT, GSK, and mTOR).

The findings in this study demonstrate that NECA regulated the mRNA expression of *PGC-1α* and *NR4A*. Moreover, the findings also demonstrated a crosstalk between NECA and insulin regarding the transcriptional level for NR4A (but not for *PGC-1α*). Therefore, modification in adenosine signal transduction pathways or adenosine concentration may lead to enhance *PGC-1α* activity and thus may have an important role in energy and oxidative metabolism in the skeletal muscle tissue. Moreover, as a crosstalk between NECA and insulin was found in terms of NR4A in this study and NR4A modulates important many biological functions, it is possible that adenosine modulation plays a potential physiological and pharmacological role in the skeletal muscle tissue.

This study has presented a cell model of skeletal muscle cells and demonstrated molecular evidence for divergent insulin-mediated alterations at multiple molecular levels of the adenosinergic signaling system, including glucose and fatty acid metabolism/insulin sensitivity as a final biological end point.

## 5. Conclusions

The findings of this study have provided evidence of the direct effects of NECA treatment *in vitro* on intracellular signaling pathways including inflammation, metabolism, and nuclear receptors demonstrating the existence of an additional signaling pathway stimulated by NECA in the rat skeletal muscle cells. A novel crosstalk between adenosine analogue and insulin has been demonstrated for the first time; evidence has been gathered *in vitro* for the effects of NECA and insulin treatment on intracellular signaling pathways, in particular glycolysis, fatty acid oxidation, and insulin sensitivity, in the skeletal muscle cells. Furthermore, the data suggest that adenosine has a novel functional role as a modulator of insulin effects on key signaling metabolic pathways in the skeletal muscle tissue. Although the findings in this study do not fully determine other downstream signaling targets such as IRS, concentration-response curves, and time-course studies for both NECA and insulin, they clearly support the possibility of employing adenosine analogues to develop new treatments for a wide range of metabolic conditions, such as diabetes and obesity.

## Figures and Tables

**Table 1:** List of gene primer and probe sequences.

Gene	Sequences (5′-3′)
NR4A1	Probe: 5′-CTTTATCCTCCGCCTGGCCTACCGA-3′ Forward primer: 5′-TGTTGCTAGAGTCCGCCTTTC-3′ Reverse primer: 5′-CAGGCCTGAGCAGAAGATGAG-3′

NR4A2	Probe: 5′-TACGCTTAGCATACAGGTCCAACCCAGTG-3′ Forward primer: 5′-CCAAAGCCGATCAGGACCT-3′ Reverse primer: 5′-GACCACCCCATTGCAAAAGAT-3′

NR4A3	Probe: 5′-ACTGTCCCACCGACCAGGCCACT-3′ Forward primer: 5′-GACGCAACGCCCAGAGAC-3′ Reverse primer: 5′-TAGAACTGCTGCACGTGCTCA-3′

PGC-1*α*	Probe: 5′-TGGAACTCTCTGGAACTGCAGGCCTAACT-3′ Forward primer: 5′-TTCCCCATTTGAGAACAAGACTATT-3′ Reverse primer: 5′-GTTATCTTGGTTGGCTTTATGAGGA-3′

CPT1B	Probe: 5′-CCTACATGATCGCAGGCGAAAACACAA-3′ Forward primer: 5′-GGCCGACCACGGATACG-3′ Reverse primer: 5′-ACTCGATAACTTGCTGGAAACATG-3′

ACC*β*	Probe: 5′-ATCGAGACGGTGCTCATCGCCAAT-3′ Forward primer: 5′-TGTCACCCGCTTTGGAGG-3′ Reverse primer: 5′-CATACACTTGACCGCAGCGAT-3′

LCAD	Probe: 5′-GGAATGAAAGCCCAGGACACAGCAGAA-3′ Forward primer: 5′-GGTGGAGAATGGAATGAAAGGAT-3′ Reverse primer: 5′-GCACTAGCTGGCAATCGAACA-3′

PDK4	Probe: 5′-CGTCGCCAGAATTAAAGCTCACACAAGTC-3′ Forward primer: 5′-AGCAGTAGTCGAAGATGCCTTTG-3′ Reverse primer: 5′-ATGTGGTGAAGGTGTGAAGGAA-3′

HKII	Probe: 5′-AGTTCCTGTCTCAGATAGAGAGCGACTGCCT-3′ Forward primer: 5′-GCATCTCAGAGCGCCTCAAG-3′ Reverse primer: 5′-GATGGCACGAACCTGTAGCA-3′

PFK	Probe: 5′-CTGCCCTGCACCGCATTGTAGAGATC-3′ Forward primer-5′-TGGCACTGATATGACCATTGGT-3′ Reverse primer-5′-TGAGCGGTGGTGGTGATG-3′

IL-6	Probe: 5′-CTCTCCGCAAGAGACTTCCAGCCAGTT-3′ Forward primer: 5′-GCCCTTCAGGAACAGCTATGA-3′ Reverse primer: 5′-TGTCAACAACATCAGTCCCAAGA-3′

TATA-BOX	Probe: 5′-TCCCAAGCGGTTTGCTGCAGTCA-3′ Forward primer: 5′-TTCGTGCCAGAAATGCTGAA-3′ Reverse primer: 5′-GTTCGTGGCTCTCTTATTCTCATG-3′

## Data Availability

The data used to support the findings of this study are available from the corresponding author upon request.
